# Hemochromatosis Gene Polymorphism as a Predictor of Sustained Virological Response to Antiviral Treatment in Egyptian Chronic Hepatitis C Patients

**DOI:** 10.5005/jp-journals-10018-1238

**Published:** 2017-09-29

**Authors:** Mai I Mehrez, Dina SA Fattah, Naglaa AA Azeem, Mohamed A Saleh, Khadiga M Mostafa

**Affiliations:** 1 Department of Hepatology, National Hepatology and Tropical Medicine Institute, Cairo, Egypt; 2 Department of Medical Biochemistry and Molecular Biology, Cairo University, Cairo, Egypt; 3 Department of Medical Biochemistry, Beni Suef University, Beni Suef, Egypt

**Keywords:** Antiviral treatment, Hemochromatosis gene polymorphism, Polymerase chain reaction.

## Abstract

**Aim:**

The aim of this article is to assess HFE C282Y gene mutations as a predictor of sustained virological response (SVR) to anti-hepatitis C virus (HCV) treatment in Egyptian patients.

**Materials and methods:**

One hundred and forty chronic hepatitis C (CHC) patients were divided into two groups: 70 patients achieved SVR and 70 patients were nonresponders (NRs). All patients were subjected to quantitative polymerase chain reaction (PCR) at baseline, 12 and 24 weeks after therapy commencement. Deoxyribonucleic acid (DNA) sequencing for HFE (C282Y) was done by restriction fragment length polymorphism PCR.

**Results:**

Sixty five patients did not have mutation and 5 patients had C282Y mutation (GA) with SVR. While 45 NRs had heterozygous C282Y mutation (GA), 4 patients (5.7%) had homozygous mutation (AA) and 21 patients (30%) had no mutation (GG). The parameters of elevated iron [transferrin saturation (TS; p < 0.001), S iron (p < 0.02), total iron binding capacity (TIBC; p < 0.001), transferrin (p < 0.016), and soluble transferrin receptor (sTfR; p-value, 0.001)] were significantly associated with C282Y mutation. However, there was no significant difference regarding ferritin values and C282Y mutation in NR patients.

**Conclusion:**

Iron overload was frequently detected in CHC patients and associated with C282Y mutation, while biochemical markers of iron overload and C282Y HFE mutation were negative prognostic factor.

**How to cite this article:** Mehrez MI, Fattah DSA, Azeem NAA, Saleh MA, Mostafa KM. Hemochromatosis Gene Polymorphism as a Predictor of Sustained Virological Response to Antiviral Treatment in Egyptian Chronic Hepatitis C Patients. Euroasian J Hepato-Gastroenterol 2017;7(2):154-157.

## INTRODUCTION

The World Health Organization has declared hepatitis C as a global health problem, with approximately 3% of the world’s population infected with HCV.^1^ Egypt has the highest prevalence of HCV in the world at 12 to 13%.^2^ Approximately 20% of blood donors are HCV Ab positive.^3^ Iron overload in the liver induces oxidative stress, which was a factor of cell membrane damage, DNA instability, and mutagenesis. Due to these effects, iron can be considered a proinflammatory, profibrogenic factor, and a potential carcinogen. Since the implementation of serological diagnostic tests for HCV identification, elevated serum iron overload indices or appearance of iron deposits in liver cells have been observed in 10 to 40% of patients with CHC and 50% of patients suffering both from CHC and hepatocellular carcinoma.^4^ Some investigations have shown an association between elevated serum iron indices or high hepatic iron concentration (HIC) and the lack of SVR in CHC patients,^5^ whereas others have shown that there is no positive correlation between HIC and decreased frequency of SVR.^6^ In 2006, Bonkovsky et al^7^ found the presence of iron in endothelial cells with triad iron score (not global iron score) as a predictor of decreased SVR. These contradictory results from different parts of the world may possibly have their source in ethnic differences and the variable polymorphisms of iron metabolism-related genes found in different populations. The aim of this work is to assess the value of HFE *C282Y* gene mutations as a predictor of SVR to antiviral treatment in Egyptian patients with CHC virus infection.

## MATERIALS AND METHODS

The study was conducted on 140 CHC patients (based on the presence of persistently elevated liver enzymes for at least 6 months and detection of HCV ribonucleic acid by PCR technique) who took antiviral treatment. The patients were divided into two groups: Group I consisted of 70 patients who achieved SVR after antiviral treatment, and group II consisted of 70 patients who did not respond to antiviral treatment (NR). Patients with malignancy, decompensated cirrhosis, hepatitis B virus coinfection, or other causes of liver disease were excluded. All patients were subjected to full history taking, clinical examination, laboratory investigations including liver and kidney biochemical profile, alfa fetoprotein (AFP), viral load, and specific tests of our study: Complete iron profile, molecular study for HFE (C282Y).

## RESULTS

The data about patient profiles are shown in Table 1. As regards age and sex, there was no statistically significant difference between SVR and NR groups (with p-value 0.140 and 0.091 respectively). Also, the whole liver profile did not show any statistically significant difference. A high statistically significant difference was observed between NR and SVR regarding GG, GA, and AA genotype, which was absent in SVR group (Table 2). Sustained virological response was associated with allele A and NR was associated with allele G (Table 2). Concerning iron status, there was statistically significant difference between SVR and NR groups regarding different parameters of iron levels [S iron, TIBC, transferrin, TS % and sTfR)]. However, no statistical significance was documented for S ferritin between the two groups (Table 3).

## DISCUSSION

Chronic hepatitis C patients have frequently elevated serum iron stores and elevated HIC, which has been associated with a poor response to interferon-alfa.^8^ The mechanism by which iron accumulates in liver infected with chronic HCV has not yet been established. Serum iron and ferritin levels were increased in patients with CHC because of their release from hepatocellular stores in association with cell necrosis.^9^ Individuals with serum iron levels in the upper range of normal as a result of genetic polymorphisms or a high iron diet may be predisposed to develop more severe chronic HCV infections.^9^ Several studies^10^ have found that heterozygous C282Y mutations are associated with hepatic iron loading in CHC patients. Iron overload seems to impair antigen-specific immune responses by decreasing the generation of T cells and by impairment of natural killer and T helper cell function. Piperno et al^11^ suggested that iron overload in patients with hemochromatosis may contribute to the persistence of HCV infection, and iron overload may in theory promote viral replication. The amount of hepatic iron has been identified as one of these factors that adversely affect the likelihood of response to interferon-alfa; those patients with higher hepatic iron content are less likely to respond to interferon therapy.^12^ In our study, there was a correlation between HFE gene mutation and iron overload. We considered transferrin saturation index (TSI) as the most specific and sensitive parameter in identifying iron overload as it showed a significant statistical difference between responder group (27.5%) and NR (34.5%) group with p-value <0.001. But there was no significant difference for serum ferritin, S iron (p = 0.02), TIBC (p > 0.001) transferrin (p = 0.016), sTfR (p > 0.001), but our study provides evidence supporting that the HFE gene mutations are associated with significant abnormalities of iron metabolism and suggests that patients with CHC accumulate iron as a result of interplay between genetic and acquired factors. We noticed that A allele is associated with higher iron parameters and lower TIBC and the homozygous mutation (AA) is associated with higher iron indices. The wildtype (GG) is lower than the heterozygous mutation (GA) genotype. There is a statistically significant difference between gene polymorphism (AA, GA, GG) and iron parameters with p-value <0.001 for each of S. iron, TIBC, TS%, and S. ferritin, with p-value 0.033 as regarding transferrin and by 0.026 as regarding sTfR. Sustained virological response rates were lower among patients with HFE gene mutations compared with those with HFE gene wildtype. In our study, 54 of 140 (38.5%) patients have mutation [50 heterozygous (GA) and 4 homozygous (AA)] and 86 have no mutation (wild-type GG). All homo and 45 from heterozygous mutation did not respond to treatment; 92% (92.9%) of the SVR group have no (GG) mutation and 7% carry C282Y mutation (GA), while 64.3% of NR group carry heterozygous C282Y mutation (GA), 5.7% carry homozygous mutation (AA), and 30% are without mutation (GG). Therefore, HFE gene mutations may act synergically with CHC in the development of liver damage, predicting a higher rate of nonresponse to therapy. Our results correlate with those of Sini et al,^13^ who stated that 69 CHC patients with end-of-treatment response were lower among patients with HFE gene mutations compared with those with HFE gene wildtype (p = 0.005) and TSI showed a significant statistical difference between HFE mutant patients (50%) and wild-type homozygotes (43.4%) (p < 0.01). Coelho-Borges et al^14^ had similar results in 2002 when they studied 44 Brazilian patients. They showed that SVR was achieved in 0 of 16 patients with HFE gene mutations and 11 (41%) of 27 patients without HFE gene mutations (p = 0.002). They concluded that heterozygosity for H63D and/ or C282Y HFE gene mutation predicted absence of SVR to combination treatment with interferon and ribavirin in patients with CHC, non-1 genotype and serum ferritin levels above 500 ng/mL. Our results did not correlate with those of Li et al,^15^ who showed that H63D mutation was associated with a significantly higher SVR rate [odds ratio (OR) = 1.60, 95% confidence interval (CI): 1.09-2.34, p = 0.020], while the C282Y mutation was not (OR = 1.19, 95% CI: 0.71-1.98, p = 0.510). We do not agree with Lebray et al^16^ who based on a large cohort of HCV-infected patients found an opposite effect of iron blood parameters and the H63D mutation on the antiviral efficacy of interferon-alfa used alone or in combination therapy with exception of six C282Y heterozygote patients that displayed no sustained response; but this group was too small to allow the detection of a significant difference with any other group. Increased iron stores may affect the course of viral infection in various ways: First, increased HIC may facilitate viral replication and *in vitro* data suggest that iron facilitates HCV replication in cultured hepatocytes.^17^ Second, iron loading was demonstrated to enhance HCV pathogenicity.^18^

**Table 1:** **Table 1:** Comparison between responder and NR group

		*Nonresponders*				*Responders*			
		*Range*		*Mean± SD*		*Range*		*Mean ±SD*	
Age (years)		20-59		43.7 ± 9.1		21-59		46.0 ± 8.8	
Blood sugar		60-266		105.5 ± 33.2		10-197		99.2 ± 24.8	
Creatinine		0.2-1.4		0.9 ± 0.2		0.6-1.4		0.9 ± 0.2	
Albumin		3.5-5.1		4.2 ± 0.4		3.5-5.7		4.1 ± 0.4	
ALP		3.7-314		133.2 ± 79.8		11-380		122.9 ± 54.2	
AST		11.7-247		60.9 ± 39.2		7-226		58.4 ± 41.8	
ALT		12-260		66.4 ± 42.4		4-195		64.6 ± 42.6	
T.Bil.		0.16-1.8		0.8 ± 0.3		0.35-1.6		0.8 ± 0.3	
D.Bil.		0.1-1.2		0.4 ± 0.3		0.1-1.1		0.4 ± 0.2	
TLC		3.1-12		6.0 ± 1.8		3.1-10.2		6.3 ± 1.8	
HB		11-18.9		14.2 ± 1.8		11-17		13.6 ± 1.4	
PLAT		112-417		212.5 ± 66.6		110-345		210.9 ± 61.2	
PC		60-100		85.2 ± 10.8		60-100		85.2 ± 10.7	
AFP		0.4-162		11.2 ± 21.0		0.5-43		6.7 ± 6.8	
S. iron (μg/dL)		38-90		56.76 ± 9.95		32-90		52.5 ± 11.1	
TIBC (μg/dL)		104-296		172.44 ± 39.72		123-410		205.3 ± 59.29	
S. ferritin (g/mL)		91-310		158.19 ± 41.32		91-274		156.37 ± 36.24	
Transferrin (mg/dL)		89-410		239.23 ± 76.95		113-340		211.23 ± 57.43	
TS %		18-61.6		34.5 ± 10.14		9-45		27.48 ± 9.32	
sTfR (nmol/L)		11.20-36		18.10 ± 6.75		11.2-18		13.91 ± 1.49	

SD: Standard deviation; ALP: Alkaline phosphatase level; AST: Aspartate aminotransferase; ALT: Analing aminotransferase; T.Bil: Total bilirubin; D.Bil: Direct bilirubin; TLC: Total leukocyte count; HB: Hemoglobin; PLAT: Plasminogen activator

**Table 2:** **Table 2:** Frequency of the genotypes of gene polymorphisms and allele frequency and OR in responder *vs* NR group

				*Nonresponders*				*Responders*							
				*Count*		*%*		*Count*		*%*		*p-value*		*OR (95% CI)*	
HFE		AA		4		5.7		0		0		0.12			
		GA		48		64.3		5		7.1		<0.001		23.4 (8.33-65.72)	
		GG		21		30		65		92.9		<0.001		0.033 (0.012-0.094)	
Allele		A		53		37.9		5		3.6		<0.001		27.414 (8.307-90.47)	
		G		87		62.1		135		96.4					

OR: Odds ratio

**Table 3:** **Table 3:** Comparison between responder and NR group as regards iron study

		*Nonresponders Mean ± SD*		*Responder Mean ± SD*		*p-value*		*Significant*	
S. iron (μ/dL)		56.76 ± 9.95		52.56 ± 11.10		0.020		S	
TIBC (μg/dL)		172.44 ± 39.72		205.30 ± 59.29		<0.001		HS	
Transferrin saturation %		34.50 ± 10.14		27.48 ± 9.32		<0.001		HS	
S. ferritin (ng/mL)		158.19 ± 41.32		156.37 ± 36.24		0.783		NS	
Transferrin (mg/dL)		239.23 ± 76.95		211.23 ± 57.43		0.016		HS	
sTfR (nmol/L)		18.10 ± 6.75		13.91 ± 1.49		<0.001		HS	

Ssignificant; NS: Nonsignificant
